# Editorial: Obesity Stigma in Healthcare: Impacts on Policy, Practice, and Patients

**DOI:** 10.3389/fpsyg.2017.02149

**Published:** 2017-12-11

**Authors:** Stuart W. Flint, Emily J. Oliver, Robert J. Copeland

**Affiliations:** ^1^Carnegie School of Sport, Leeds Beckett University, Leeds, United Kingdom; ^2^Department of Sport and Exercise Sciences, Durham University, Durham, United Kingdom; ^3^Centre of Sport and Exercise Science, Sheffield Hallam University, Sheffield, United Kingdom

**Keywords:** weight stigma, healthcare, higher weight, anti-fat attitudes, weight bias

## Introduction and edition purpose

Obesity prevalence is a global health concern. Alongside increasing awareness of the condition are concomiteted increases in reported weight stigma and discrimination toward people with obesity. Counter-intuitively, research has identified weight stigma in settings that are critical for the engagement and treatment of people with obesity, such as exercise (Robertson and Vohora, 2008; Flint and Reale, 2016), healthcare facilities (Brown and Flint, 2013), schools (Puhl and Luedicke, 2012), and workplaces (Roehling, 1999; Flint et al., 2016). The prevalence and robustness to interventions to reduce anti-fat attitudes is concerning (Flint et al., 2013) given their association with anti-fat behavior (O'Brien et al., 2008). For instance, healthcare professionals and students in training report stigmatizing attitudes and beliefs toward higher-weight people, and in some cases, withhold appropriate advice or treatment (e.g., Kristeller and Hoerr, 1997; Hebl and Xu, 2001). In addition, healthcare providers use stigmatizing terminology in consultations and other patient-practitioner meetings, with adverse effects (e.g., avoidance of healthcare settings, and compromised psychosocial wellbeing: depressed mood, anxiety, social isolation, and lower self-esteem) (e.g., Vartanian and Novak, 2011).

Despite accumulating evidence demonstrating prevalent weight stigma in healthcare settings, current knowledge of the *impact* of weight stigma within healthcare remains underdeveloped. In a dynamic context whereby the legal and social standing of higher weight people is the subject of contemporary debate, an evidence-based review of understanding and practice is timely. Existing research has provided useful and critical insight into the prevalence, breadth and nature of anti-fat biases and weight stigma within healthcare professionals and settings. Increasingly, we are aware of how these biases might influence treatment and the patient experience. Conceptualizing existing research as predominantly addressing these 'first generation' questions, in editing the current Research Topic, we sought to present emerging work that explores second and third generation questions. These concern, for example, differential predictors of patients' reactivity to weight stigma, new interventions for modification of weight self-stigma, and theoretically-grounded critical reflections on how stigma is experienced and socially constructed. We include work from all stages of the healthcare pathway, exploring: whether and how theory and evidence concerning weight stigma are reflected in policy and guidance, how stigma is influencing professionals and their practice, and how patients are affected.

## Summary of contributing articles

This Research Topic opens with Lee and Pause's auto-ethnographic account of fat stigma and discrimination that people experience from the medical profession and other sectors of the community. Novel contributions are made through the authors' consideration of Bacon and Aphramor's “Health and Every Size” paradigm as a path to health for individuals who are fat, raising critical questions concerning the nature of health as a state, behavior, commodity, or social contract. Importantly, this article presents research into the barriers to accessing and adoption of health behaviors from the perspective of higher weight researchers. In doing so, the authors consider whether the “Health at every Size” paradigm is an appropriate health perspective that higher weight people can utilize. Drawing from feminist theory, the authors challenge a perceived failure to provide evidenced-based healthcare to higher weight people.

The second paper (Rudolph and Hilbert) presents an experimental examination of the impact of obesity-related health messages on implicit and explicit weight bias. Rudolph and Hilbert's study examined the use of health messages promoting healthy eating and physical activity on subsequent implicit and explicit weight bias comparing the findings against a control arm that contained neutral information. The authors reported a small difference in reduced implicit weight bias in the experimental condition (health messages) but not in the control condition (neutral messages). Despite this positive finding, there was no difference in explicit weight bias. Given the commonality of health messages, further research that examines the implications on weight bias appears warranted.

The third paper (Meadows et al.) explored the effects of both the amount of contact with higher weight people before and during medical school and of training to induce empathy toward patients on anti-fat attitudes. An online survey was completed by students in their first year and again in their fourth year of medical school. After 4 years of medical school, greater contact with higher weight patients improved attitudes toward higher weight patients, however, this effect was not as strong for attitudes toward higher weight people. Differing effects were reported for the impact of training to include empathy toward patients, where greater effect was observed for participants who were more egalitarian and empathetic at baseline. This study along with other interventions (e.g., Flint et al., 2013) that have shown only a small effect in reducing weight stigma, reinforce the need for interventions to improve attitudes toward higher weight people.

The fourth study (Raves et al.) used a mixed-methods design (survey responses, ethnographic data and multi-year participant-observations within a clinical setting) to examine the relationship between weight stigma and post-surgical dietary response; whether weight loss reduces weight stigma; and patient and provider perspectives on stigma and healthcare adherence. Raves et al. reported that weight stigma internalization and experiences of weight stigma predicted worse dietary adherence; patients were ambivalent of the stigma to adherence relationship, whereas healthcare professionals viewed this as poor patient compliance. This study provides evidence of weight stigma in healthcare, and that internalization and experiences of weight stigma reduces healthcare adherence, highlighting the need for intervention to improve adherence and potentially outcomes.

The final two articles discuss the use of terminologies and labels used in policy, research, healthcare and other contexts. First, Lozano-Sufrategui et al. discuss the terminology used by the National Institute for Health and Care Excellence in England within the national guidance for improving health and social care in England given the status of NICE in shaping the discourse relating to obesity. Second, Meadows and Daníelsdóttir suggest that more neutral terms such as “weight” and “higher weight” be used as more neutral and acceptable terms that carry less culturally constructed values.

## Emergent recommendations

Collectively, the work in this special issue underpins a number of recommendations. First, we recommend that clinicians, researchers, health practitioners, exercise specialists and policy makers carefully avoid labeling higher weight patients with culturally stigmatizing terminology. While there may be diagnostic settings where more specific terminology is required, we support calls in this Research Topic for healthcare professionals to understand what terms are acceptable for their patients. For instance, in some cases the use of “higher weight” or “fat” might be acceptable for patients. It is therefore imperative that healthcare professionals establish the most acceptable terms to use with their patients to avoid potential disengagement and associated implications for the patient-practitioner relationship. Second, we call for researchers to develop effective and innovative interventions to sustainably reduce weight stigmatizing attitudes and practices. Work thus far is dominated by acute experimental studies; more translational research into practice-focused interventions is required. Third, and finally, that research and policy makers consider resources for engaging and supporting higher weight people and mandatory training of practitioners through a stigma-awareness raising lens, given the potential impact of these on the patient healthcare outcomes.

## Author contributions

SF, EO, and RC drafted, revised and finalized the content of the manuscript. All authors have read and approved the final manuscript.

### Conflict of interest statement

The authors declare that the research was conducted in the absence of any commercial or financial relationships that could be construed as a potential conflict of interest.
